# Influence of probable respiratory sarcopenia on chronic lung diseases: a population-based cohort study of community-dwelling Chinese older adults

**DOI:** 10.3389/fmed.2025.1617808

**Published:** 2025-08-19

**Authors:** Haixia Yu, Kangkang Chen, Qifeng Chen, Laichao Xu

**Affiliations:** ^1^Department of Nursing, Affiliated Hospital of Shaoxing University, Shaoxing, China; ^2^Department of Non-communicable Diseases Control and Prevention, Shaoxing Center for Disease Control and Prevention, Shaoxing, China; ^3^Administrative Office, Shaoxing Center for Disease Control and Prevention, Shaoxing, China

**Keywords:** CHARLS, chronic lung diseases, longitudinal investigations, older adults, respiratory sarcopenia

## Abstract

**Background and objectives:**

The impact of probable respiratory sarcopenia (RS) on the prevalence and incidence of chronic lung diseases (CLDs) in middle-aged and older adults remains poorly understood. This study utilized data from the China Health and Retirement Longitudinal Study (CHARLS) to explore this association.

**Methods:**

A total of 6,614 participants aged 45 and above were included in a cross-sectional analysis in 2011, and 5,630 participants were followed for 7 years for longitudinal analysis. Probable RS was defined as a coexistence of low respiratory muscle strength and reduced appendicular skeletal muscle (ASM) mass by a position paper by four professional organizations. CLDs were identified based on self-reported medical diagnoses, including asthma, chronic bronchitis, emphysema, and pulmonary heart disease. Statistical analyses included logistic and Cox proportional hazards regression models to assess the association between probable RS and CLDs, adjusted for a wide range of covariates.

**Results:**

In the cross-sectional analysis, probable RS [odds ratio (OR) = 2.18, 95% confidence interval (CI) = 1.84 ~ 2.58, *p <* 0.001], low ASM mass (OR = 1.79, 95% CI = 1.51 ~ 2.11, *p <* 0.001), and low respiratory muscle strength (OR = 2.76, 95% CI = 2.14 ~ 3.55, *p <* 0.001) were significantly associated with increased CLDs prevalence. In the longitudinal analysis, probable RS [hazard ratio (HR) = 1.49, 95% CI = 1.26 ~ 1.77, *p <* 0.001], low ASM mass (HR = 1.47, 95% CI = 1.25 ~ 1.73, *p <* 0.001), and low respiratory muscle strength (HR = 1.31, 95% CI = 1.09 ~ 1.57, *p =* 0.004) were associated with increased CLDs incidence.

**Conclusion:**

Probable RS significantly influences the prevalence and development of CLDs among middle-aged and older adults. Early identification and targeted interventions to mitigate RS may reduce CLDs burden in this population.

## Introduction

Chronic lung diseases (CLDs) are a collection of chronic bronchitis, emphysema, pulmonary heart disease, and other related conditions. In 2017, CLDs affected 7.4% of the global population, and were responsible for 7.0% of all deaths (1). Due to factors such as aging demographics (2), it is estimated that number of global deaths and years of life lost resulted from CLDs were projected to increase by more than 1.6 million and 15 million, respectively, from 2016 to 2040 (3). Additionally, CLDs were not only associated with high mortality rates but also led to substantial healthcare utilization and decreased quality of life (4, 5), posing significant health challenges globally.

Recent studies have established a link between sarcopenia—an age-related decline in skeletal muscle mass and function—and various noncommunicable diseases such as cardiovascular diseases (6), cognitive dysfunction (7), and chronic kidney disease (8). Wang et al. (9) further identified an association between sarcopenia and the development of CLDs in the general population. However, most existing studies, including Wang et al.’s work, have primarily focused on the systemic manifestations of sarcopenia. Emerging evidence suggested that respiratory muscle weakness may also occur independently of systemic sarcopenia (10). To study this condition systematically, a term “respiratory sarcopenia (RS)” emerged during a discussion on sarcopenia. After iterating through multiple versions (11, 12), four leading groups—the Japanese Society for Respiratory Care and Rehabilitation, Japanese Association on Sarcopenia and Frailty, Japanese Society of Respiratory Physical Therapy, and Japanese Association of Rehabilitation Nutrition—collaborated to publish a position paper in 2023 (13). This document offered the most accepted definition of RS as a coexistence of reduced respiratory muscle strength and loss of respiratory muscle mass. Recognizing the challenges in quantitatively assessing respiratory muscle mass in clinical settings due to insufficient data (14), the paper introduced the idea of probable RS (13). This was characterized by low respiratory muscle strength plus a decrease in appendicular skeletal muscle mass (ASM), particularly if respiratory muscle mass was difficult to measure.

Since older adults are naturally susceptible to both respiratory muscle degeneration and CLDs vulnerabilities, these conditions are widely prevalent in this age group. So far, the clinical values of probable RS in the prevalence and incidence of CLDs remains poorly understood and under-researched, especially in population-based settings. We therefore conducted cross-sectional and prospective analyses to fill this gap on the basis of a large cohort of community-dwelling middle-aged and older adults in China. Additionally, as probable RS was a substitute for RS whose clinical values have been unfamiliar to us, our study also provides an new insight into the impact of RS on CLDs.

## Methods

### Study participants

Our study’s participants were selected from The China Health and Retirement Longitudinal Study (CHARLS) datasets. Launched in 2011, CHARLS is an ongoing cohort study that captures a nationally representative sample of Chinese adults aged 45 and older, spanning 450 villages in 150 counties across 28 provinces. The initial wave surveyed 17,708 participants, with subsequent follow-ups carried out every two or 3 years. The study gathered a wide range of data, including demographics, health status and functions, biochemical indicators, body measurements, and other relevant factors. Detailed descriptions of CHARLS are available in prior publications (15). The protocol was approved by the Ethical Review Committee of Peking University (IRB00001052-11015). All participants provided written informed consent before the examination.

**Figure 1:**
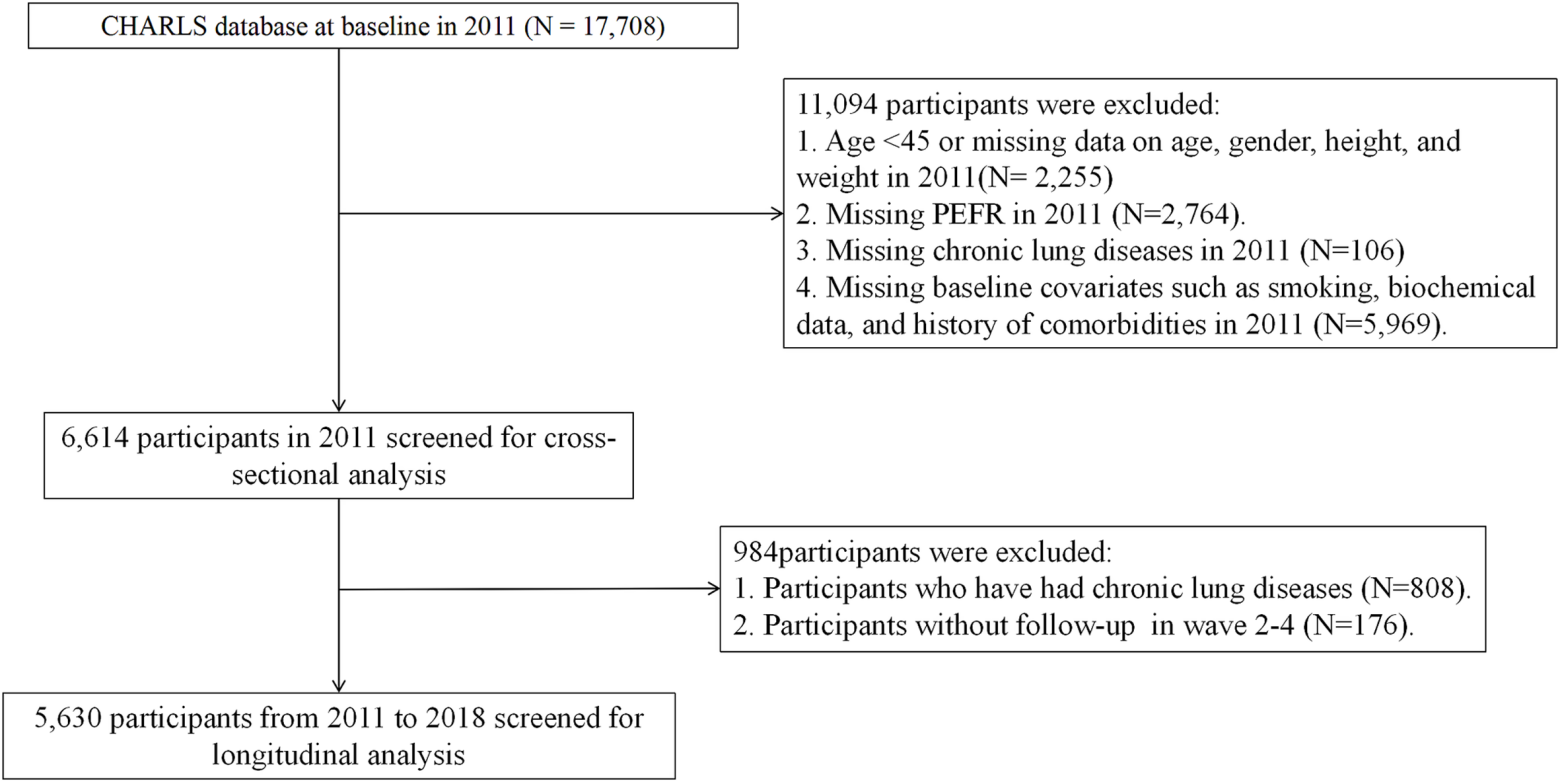
Flowchart of the sample selection process.

As of now, 5 waves of data have been made available by the CHARLS project, with releases in 2011 (wave 1), 2013 (wave 2), 2015 (wave 3), 2018 (wave 4), and 2020 (wave 5). Since the wave 5 was conducted after the Corona Virus Disease 2019 (COVID-19) epidemic, our analyses were limited to the wave 1 ~ 4. To diagnose baseline probable RS (as outlined in the following paragraph) in adults, participants in wave 1 aged <45 years or those lacking essential data, including age, gender, height, weight, and peak expiratory flow rate (PEFR), were excluded. For the multivariable analyses, participants with missing data on CLDs or key covariates such as smoking status, biochemical variables, and comorbidity history in wave 1 were also excluded. Ultimately, 6,614 participants were qualified for the cross-sectional analysis; additionally, for the longitudinal analysis, we excluded those with CLDs recorded in 2011 or who were not followed up at all in waves 2 ~ 4, resulting in 5,630 participants being eligible for a 7-year longitudinal analysis. The detailed screening process is presented in Figure 1.

### Peak expiratory flow rate measurement

For PEFR measurement, participants were instructed to stand upright, take a maximal inhalation, and exhale forcefully into a peak flow meter (Everpure™ Peak Flow Meter, Shanghai, China) through a disposable mouthpiece. The procedure was repeated three times, and the highest of the three readings was used for analysis. The predicted PEFR for men was calculated as: PEFR (L/s) = 21.566 + 0.078 × H −6.192 × ln(A), and for women: PEFR (L/s) = −22.741 + 6.901 × ln(H) −0.090 × A, where A represents age in years and H represents height in centimeters (16). These equations were developed on the basis of Chinese older adults from Jinan city, which closely matches the characteristics of our study population, thereby minimizing demographic bias. PEFR (% predicted) was calculated as (measured PEFR/predicted PEFR) × 100%. A PEFR (% predicted) <80% was defined as indicative of low respiratory muscle strength (14).

### Muscle mass measurement

Because CHARLS did not provide direct measurements of muscle mass, appendicular skeletal muscle (in kg) was estimated using an anthropometric formula validated in multiple studies (17–19), which demonstrated strong agreement with dual-energy X-ray absorptiometry (DXA). The formula was as follows:

Appendicular Skeletal Muscle = 0.193 × weight (kg) + 0.107 × height (cm) −4.157 × sex −0.037 × age (years) − 2.631, where sex was coded as 1 for men and 2 for women.

To align with the 2019 Asian Working Group for Sarcopenia (AWGS) diagnostic criteria, we further adjusted appendicular skeletal muscle for height squared (in meters) to derive appendicular skeletal muscle mass (ASM, kg/m^2^). Low ASM was defined as <7.0 kg/m^2^ for men and <5.4 kg/m^2^ for women, consistent with AWGS recommendations (20).

### Definition of probable respiratory sarcopenia

According to the diagnostic criteria proposed in the 2023 position paper (13), probable RS was defined as the coexistence of reduced respiratory muscle strength and decreased skeletal muscle mass. In this study, reduced respiratory muscle strength was identified as a PEFR <80% of the predicted value, and low skeletal muscle mass was defined as ASM <7.0 kg/m^2^ for men and <5.4 kg/m^2^ for women.

### Determination of chronic lung diseases

CLDs status was defined as a response of “Yes” to either of the following two questions: “Has your doctor ever told you that you have chronic lung diseases, such as chronic bronchitis or emphysema, or even pulmonary heart disease (excluding tumors or cancer)?” or “Has your doctor ever told you that you have asthma?” This self-report method is commonly adopted in large-scale epidemiological studies where objective clinical testing is not feasible.

### Covariates

The selection of covariates was based on their relevance to probable RS and CLDs, as well as the availability of data. These included marital status, education level, residential area, smoking status, alcohol use, physical activities, diastolic blood pressure (DBP), C-reactive protein (CRP), glycated hemoglobin (HbA1c), triglycerides, non-high-density lipoprotein cholesterol (NonHDL-C), glucose, and common comorbidities such as hypertension, diabetes, cancer, heart diseases, stroke, dyslipidemia, digestive diseases, psychiatric problems, and rheumatism. To avoid multicollinearity, we did not include age, gender, weight, height, or BMI as covariates in any models involving ASM or probable RS, as these variables were directly used in the ASM estimation formula.

### Statistical analysis

We present the baseline characteristics of participants using either numbers (percentages) or means±standard deviations, according to their CLDs status. Statistical differences were compared by the chi-square test or independent *t* test as appropriate. Associations between CLDs prevalence and probable RS (yes/no), low ASM mass (low/normal to high), and low PEFR (low/normal to high) were examined cross-sectionally in 2011, using individuals without CLDs as controls. Multivariable logistic regression models were adjusted for covariates that showed *p* < 0.10 in univariate analyses to minimize overfitting while retaining potentially relevant confounders. Adjusted odds ratios (ORs) and 95% confidence intervals (CIs) were reported. With respect to the longitudinal trends, we applied a life table method stratified by probable RS and its components (low ASM mass and low PEFR) to track CLDs development over 7 years of follow-up. The log-rank test determined the incidence equality among groups, while Cox proportional hazards regression analyzed the impact of each factor on new CLD cases, considering potential confounders that showed *p* < 0.10 in univariate analyses. The proportional hazards assumption was assessed using Schoenfeld residuals. We also performed subgroup analyses to further assess interactions between probable RS and potential confounders, which made older adults more prone to develop CLD. Data processing and analysis were performed using R version 4.3.0. All tests were 2-tailed, and *p* < 0.05 was considered statistically significant.

## Results

### A cross-sectional analysis in 2011

In 2011, a total of 6,614 eligible participants were included in the cross-sectional analysis, of whom 808 (12.2%) reported having CLDs. Table 1 presents the baseline characteristics on the basis of CLDs status. Older age, women, unmarried/divorced/widowed status, low educational level, rural residence, smoking, alcohol consumption, lack of physical activity, low BMI, elevated CRP levels, low triglycerides, and the presence of pre-existing conditions such as heart diseases, stroke, digestive diseases, psychiatric disorders, or rheumatism were significantly more prevalent in community-dwelling adults with CLDs. Additionally, adults with probable RS (*p* < 0.001) or its components (low ASM and low PEFR, both *p* < 0.001) were also more likely to suffer from CLDs in relation to those without these conditions. To further examine the relationship between PEFR and ASM, we calculated their pairwise correlation. A moderate positive correlation was observed (*r* = 0.425, 95% CI = 0.405–0.445, *p* < 0.001), as shown in Supplementary Figure S1, indicating that while these two variables are interrelated, they reflect distinct physiological attributes.

**Table 1:** Baseline characteristics according to chronic lung disease status in a population-based sample of Chinese adults aged ≥45.

Variables	In 2011			After 7 years of follow-up		
	Chronic lung diseases (no, *n* = 5,806)	Chronic lung diseases (yes, *n* = 808)	*P*	Chronic lung diseases (no, *n* = 4,859)	Chronic lung diseases (yes, *n* = 771)	*P*
Age, mean (SD), years	59.75 ± 9.62	63.48 ± 9.49	**<0.001**	59.37 ± 9.54	60.74 ± 9.26	**<0.001**
Sex, n (%)			**<0.001**			**0.002**
Women	2,676 (46.09)	464 (57.43)		2,187 (45.01)	394 (51.10)	
Men	3,130 (53.91)	344 (42.57)		2,672 (54.99)	377 (48.90)	
Marital status, n (%)			**0.006**			0.051
Married/married but separated	5,073 (87.38)	678 (83.91)		4,274 (87.96)	659 (85.47)	
Unmarried/divorced/widowed	733 (12.62)	130 (16.09)		585 (12.04)	112 (14.53)	
Education level, n (%)			**0.004**			0.076
Junior and below	5,232 (90.11)	754 (93.32)		4,362 (89.77)	708 (91.83)	
Senior and above	574 (9.89)	54 (6.68)		497 (10.23)	63 (8.17)	
Residential area, n (%)			**0.002**			**<0.001**
Urban	2,079 (35.81)	245 (30.32)		1,766 (36.34)	231 (29.96)	
Rural	3,727 (64.19)	563 (69.68)		3,093 (63.66)	540 (70.04)	
Smoking status, n (%)			**<0.001**			**<0.001**
No	3,582 (61.69)	380 (47.03)		3,064 (63.06)	430 (55.77)	
Yes	2,224 (38.31)	428 (52.97)		1,795 (36.94)	341 (44.23)	
Alcohol consumption, n (%)			**<0.001**			0.084
No	3,563 (61.37)	446 (55.20)		3,007 (61.89)	452 (58.63)	
Yes	2,243 (38.63)	362 (44.80)		1,852 (38.11)	319 (41.37)	
Physical activities, n (%)			**0.027**	4,555 (93.74)	748 (97.02)	**<0.001**
No	5,473 (94.26)	777 (96.16)		304 (6.26)	23 (2.98)	
Yes	333 (5.74)	31 (3.84)				
BMI, mean (SD), kg/m^2^	23.47 ± 3.65	22.59 ± 3.99	**<0.001**	23.57 ± 3.60	23.08 ± 3.88	**0.001**
DBP, n (%)			0.112			0.894
<90 mmHg	5,105 (87.93)	726 (89.85)		4,271 (87.90)	679 (88.07)	
≥90 mmHg	701 (12.07)	82 (10.15)		588 (12.10)	92 (11.93)	
CRP, n (%)			**<0.001**			**0.006**
<3 mg/L	4,581 (78.90)	577 (71.41)		3,888 (80.02)	584 (75.75)	
≥3 mg/L	1,225 (21.10)	231 (28.59)		971 (19.98)	187 (24.25)	
Triglycerides, n (%)			**0.034**			0.774
<150 mg/dL	4,221 (72.70)	616 (76.24)		3,524 (72.53)	563 (73.02)	
≥150 mg/dL	1,585 (27.30)	192 (23.76)		1,335 (27.47)	208 (26.98)	
NonHDL cholesterol, n (%)			0.290			0.112
<160 mg/dL	4,084 (70.34)	583 (72.15)		3,445 (70.90)	525 (68.09)	
≥160 mg/dL	1,722 (29.66)	225 (27.85)		1,414 (29.10)	246 (31.91)	
Glucose, n (%)			0.337			0.380
<100 mg/dL	2,326 (40.06)	338 (41.83)		1,942 (39.97)	321 (41.63)	
≥100 mg/dL	3,480 (59.94)	470 (58.17)		2,917 (60.03)	450 (58.37)	
Hypertension, n (%)			0.234			0.058
No	4,247 (73.15)	575 (71.16)		3,586 (73.80)	544 (70.56)	
Yes	1,559 (26.85)	233 (28.84)		1,273 (26.20)	227 (29.44)	
Diabetes, n (%)			0.721			0.894
No	5,430 (93.52)	753 (93.19)		4,550 (93.64)	721 (93.51)	
Yes	376 (6.48)	55 (6.81)		309 (6.36)	50 (6.49)	
Cancer, n (%)			0.484			0.187
No	5,762 (99.24)	800 (99.01)		4,829 (99.38)	762 (98.83)	
Yes	44 (0.76)	8 (0.99)		30 (0.62)	9 (1.17)	
Heart diseases, n (%)			**<0.001**			**<0.001**
No	5,130 (88.36)	643 (79.58)		4,334 (89.20)	641 (83.14)	
Yes	676 (11.64)	165 (20.42)		525 (10.80)	130 (16.86)	
Stroke, n (%)			**0.024**			0.394
No	5,662 (97.52)	777 (96.16)		4,745 (97.65)	749 (97.15)	
Yes	144 (2.48)	31 (3.84)		114 (2.35)	22 (2.85)	
Dyslipidemia, n (%)			0.472			0.592
No	5,235 (90.17)	735 (90.97)		4,391 (90.37)	692 (89.75)	
Yes	571 (9.83)	73 (9.03)		468 (9.63)	79 (10.25)	
Digestive diseases, n (%)			**<0.001**			**<0.001**
No	4,534 (78.09)	568 (70.30)		3,867 (79.58)	524 (67.96)	
Yes	1,272 (21.91)	240 (29.70)		992 (20.42)	247 (32.04)	
Psychiatric problems, n (%)			**0.037**			0.538
No	5,741 (98.88)	792 (98.02)		4,808 (98.95)	761 (98.70)	
Yes	65 (1.12)	16 (1.98)		51 (1.05)	10 (1.30)	
Rheumatism, n (%)			**<0.001**			**<0.001**
No	3,812 (65.66)	430 (53.22)		3,299 (67.89)	396 (51.36)	
Yes	1,994 (34.34)	378 (46.78)		1,560 (32.11)	375 (48.64)	
Low ASM (%)			**<0.001**			**<0.001**
No	4,502 (77.54)	520 (64.36)		3,843 (79.09)	550 (71.34)	
Yes	1,304 (22.46)	288 (35.64)		1,016 (20.91)	221 (28.66)	
Low PEFR (%)			**<0.001**			**0.001**
No	1,310 (22.56)	72 (8.91)		1,136 (23.38)	140 (18.16)	
Yes	4,496 (77.44)	736 (91.09)		3,723 (76.62)	631 (81.84)	
Probable RS (%)			**<0.001**			**<0.001**
No	4,736 (81.57)	537 (66.46)		4,029 (82.92)	588 (76.26)	
Yes	1,070 (18.43)	271 (33.54)		830 (17.08)	183 (23.74)	

RS, Respiratory Sarcopenia; SD, stand deviation; PEFR, peak expiratory flow rate; ASM, appendicular skeletal muscles; BMI, body mass index; DBP, diastolic blood pressure; CRP, C-reactive protein; HbA1c, glycated hemoglobin; HDL-C, high-density lipoprotein cholesterol. Bold values indicate statistically significant results (*P* < 0.05).

**Figure 2:**
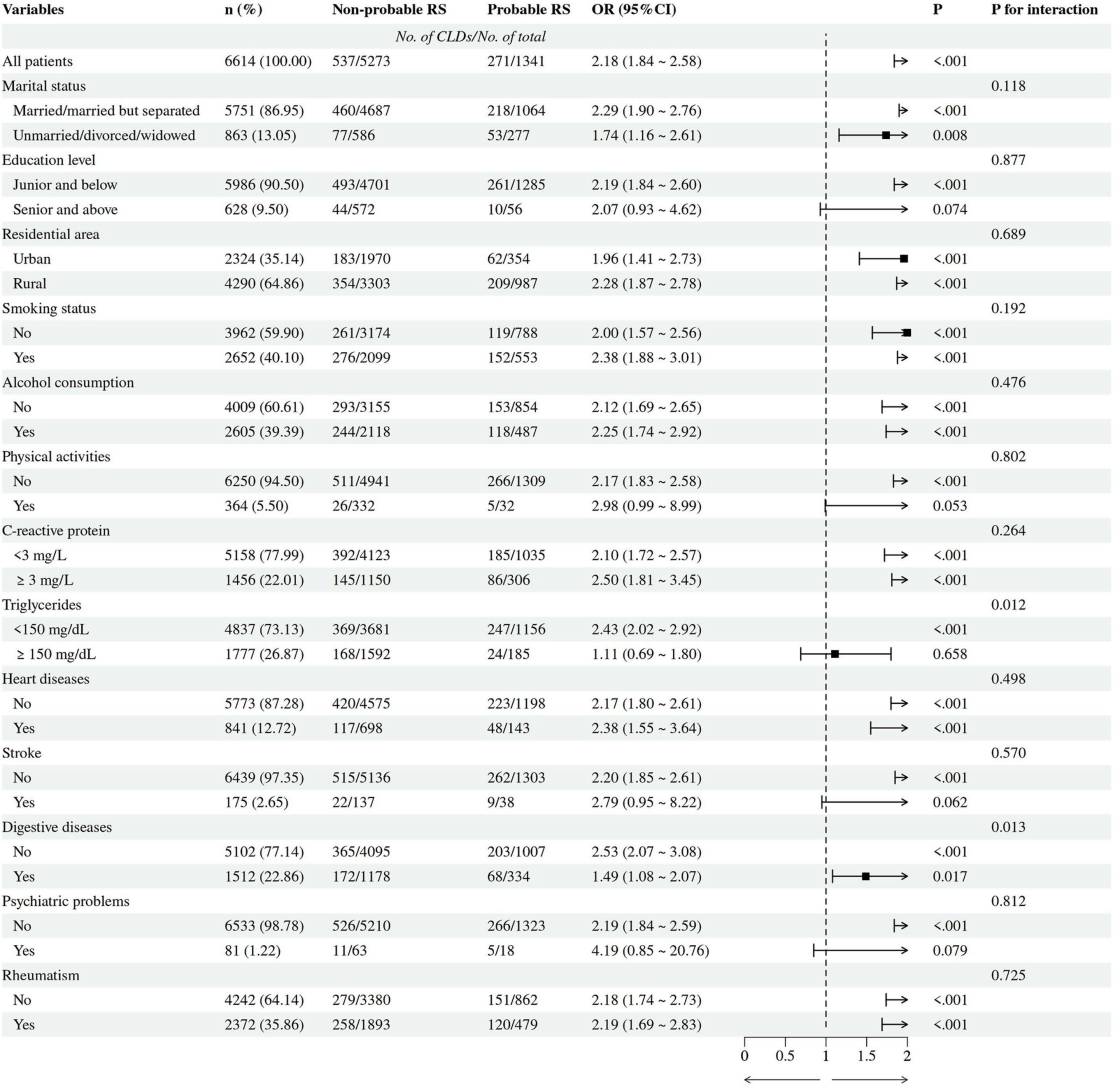
The influences of the interactions between probable RS and potential confounders on prevalence of chronic lung diseases (*N* = 6,614).

When putting potential covariates (marital status, education level, residential area, smoking status, alcohol consumption, physical activities, CRP, triglycerides, heart problems, stroke, digestive disease, psychiatric problems, rheumatism) into logistic regression models, we observed that the associations of low ASM mass (OR = 1.79, 95% CI = 1.51 ~ 2.11, *p <* 0.001), low PEFR (OR = 2.76, 95% CI = 2.14 ~ 3.55, *p <* 0.001), and probable RS (OR = 2.18, 95% CI = 1.84 ~ 2.58, *p <* 0.001) with an increased prevalence of CLDs remained significant (Table 2). Subgroup analyses were also conducted to assess the impacts of interactions between probable RS and potential confounders on the prevalence of CLDs (Figure 2). These analyses indicated that probable RS may specifically increase the risk of CLDs in community-dwelling adults with low triglyceride levels (*P* for interaction = 0.012) and in those without digestive diseases (*P* for interaction = 0.014).

**Table 2:** Logistic model for prevalence of chronic lung diseases in 2011, according to ASM mass, PEFR and probable RS (*N* = 6,614).

Variables	Model 1		Model 2	
	OR (95% CI)	*P*	OR (95% CI)	*P*
ASM mass (ref. normal to high)	1.79 (1.51 ~ 2.11)	**<0.001**	**—**	**—**
PEFR (ref. normal to high)	2.76 (2.14 ~ 3.55)	**<0.001**	**—**	**—**
Probable RS (ref. no)	**—**	**—**	2.18 (1.84 ~ 2.58)	**<0.001**
Marital status (ref. married/married but separated)	1.17 (0.95 ~ 1.45)	0.146	1.16 (0.94 ~ 1.44)	0.162
Education level (ref. junior and below)	0.84 (0.62 ~ 1.14)	0.267	0.81 (0.60 ~ 1.09)	0.164
Residential area (ref. rural area)	1.15 (0.97 ~ 1.36)	0.117	1.12 (0.95 ~ 1.33)	0.177
Smoking status (ref. no)	1.81 (1.52 ~ 2.14)	**<0.001**	1.82 (1.53 ~ 2.15)	**<0.001**
Alcohol consumption (ref. no)	1.07 (0.90 ~ 1.27)	0.445	1.07 (0.90 ~ 1.27)	0.459
Physical activities (ref. no)	0.90 (0.60 ~ 1.34)	0.605	0.84 (0.56 ~ 1.25)	0.381
CRP (ref. <3 mg/L)	1.40 (1.18 ~ 1.67)	**<0.001**	1.42 (1.19 ~ 1.68)	**<0.001**
Triglycerides (ref. <150 mg/dL)	0.93 (0.77 ~ 1.11)	0.412	0.93 (0.78 ~ 1.12)	0.454
Heart problems (ref. no)	1.88 (1.54 ~ 2.30)	**<0.001**	1.91 (1.57 ~ 2.33)	**<0.001**
Stroke (ref. no)	1.23 (0.81 ~ 1.85)	0.326	1.27 (0.84 ~ 1.92)	0.249
Digestive disease (ref. no)	1.31 (1.10 ~ 1.55)	**0.002**	1.32 (1.11 ~ 1.57)	**0.002**
Psychiatric problems (ref. no)	1.33 (0.75 ~ 2.38)	0.329	1.39 (0.78 ~ 2.48)	0.259
Rheumatism (ref. no)	1.58 (1.35 ~ 1.85)	**<0.001**	1.58 (1.35 ~ 1.84)	**<0.001**

ASM, appendicular skeletal muscles; PEFR, peak expiratory flow rate; RS, respiratory sarcopenia; CPR, C-reactive protein; OR, odds ratio; CI, confidence intervals. Model 1: ASM mass + PEFR + Covariates. Model 2: probable RS + Covariates. Bold values indicate statistically significant results (*P* < 0.05).

### A 7-year longitudinal analysis

After an average of 7 years of follow-up, 5,630 adults participated in at least one reexamination (85.1% response rate). Of those, 771 (13.7%) developed CLDs over the follow-up period. Table 1 presents the baseline characteristics of participants, stratified by the incidence of CLDs. Briefly, the distribution differences was almost similar to those in the cross-sectional analysis.

**Figure 3:**
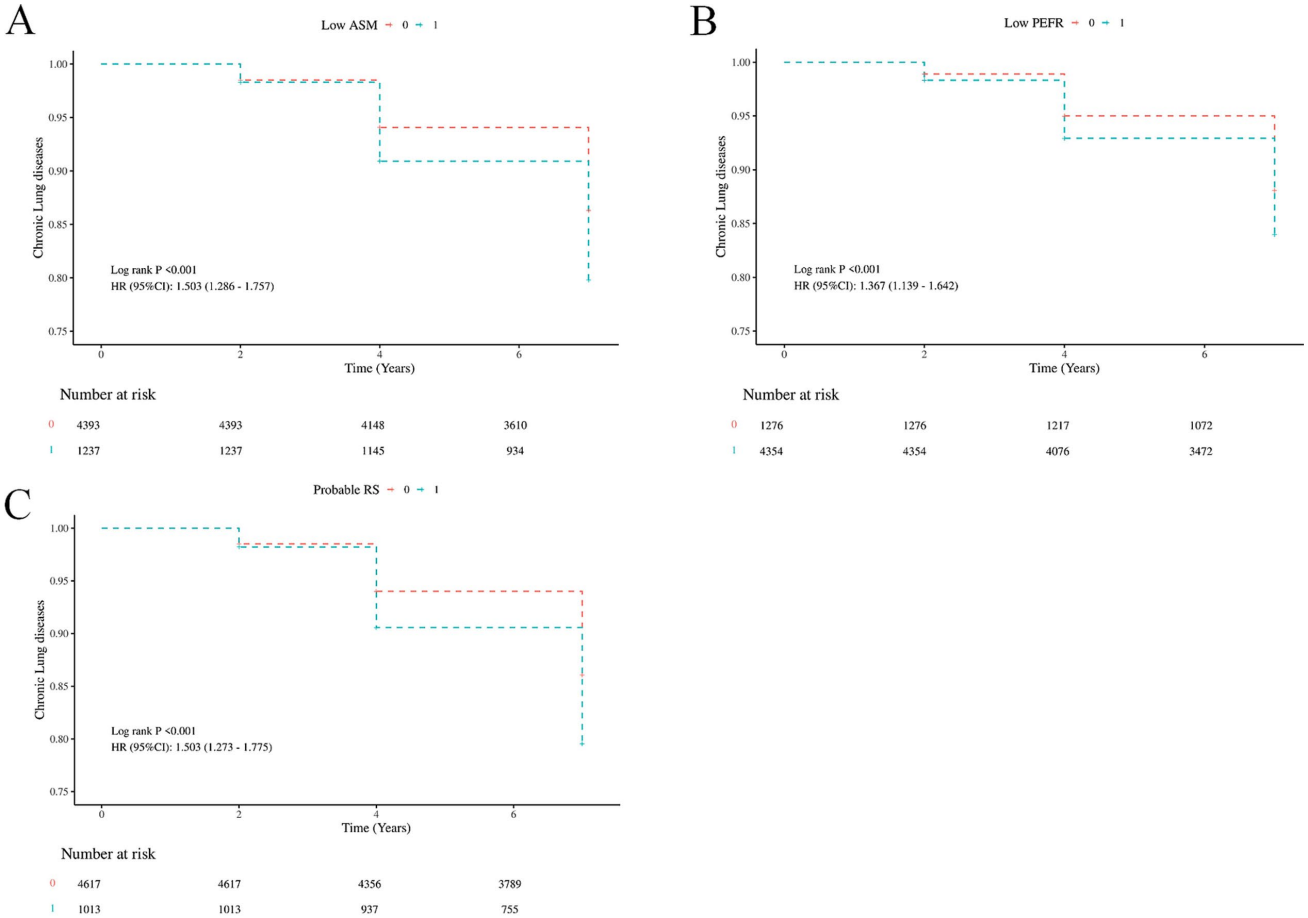
Life table survival curves exploring the association of incident chronic lung diseases with **(A)** ASM mass, **(B)** PEFR, and **(C)** probable RS. ASM, appendicular skeletal muscle; PEFR, peak expiratory flow rate; RS, respiratory sarcopenia).

**Figure 4:**
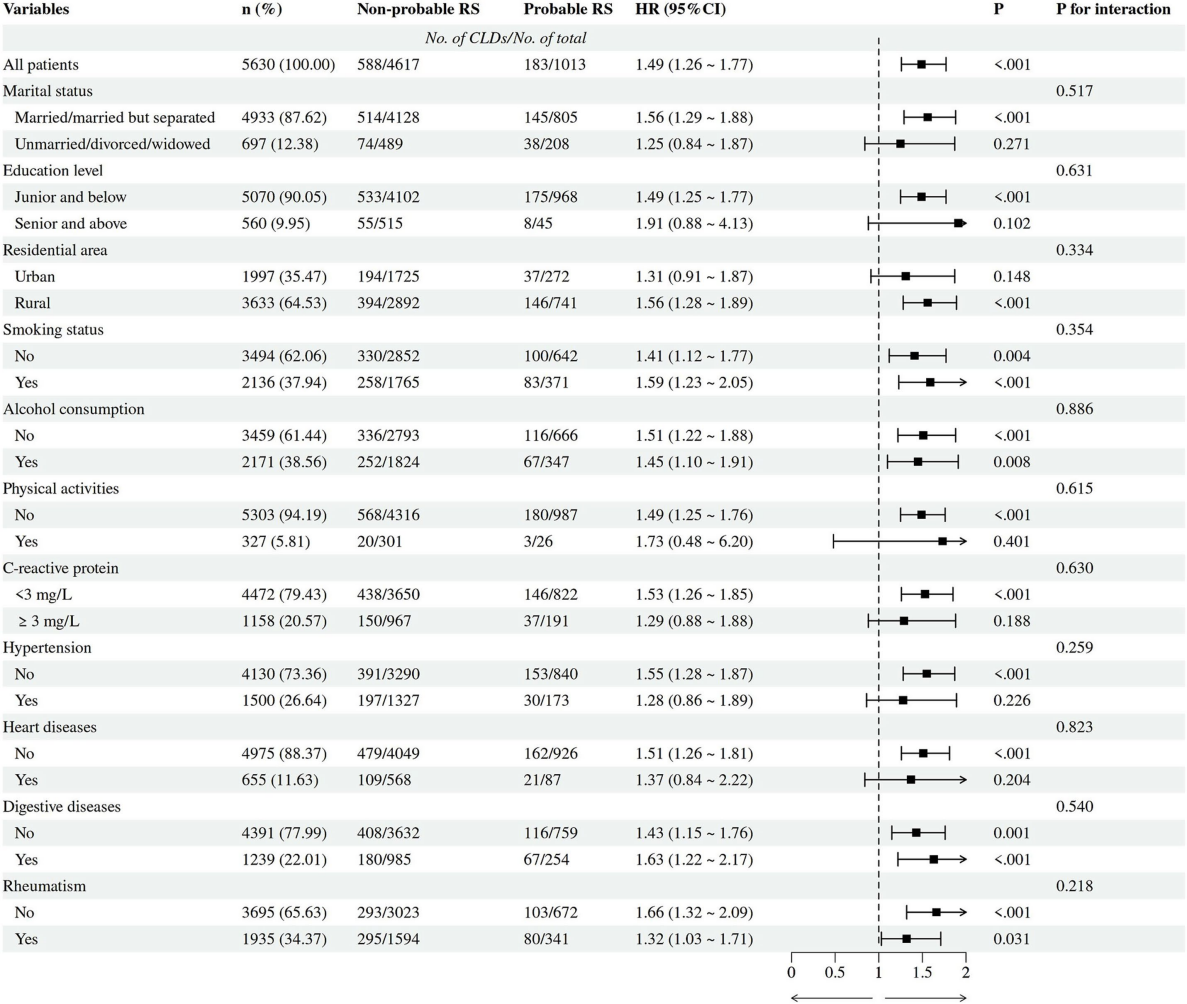
The influences of the interactions between probable RS and potential confounders on 7-year incidence of chronic lung diseases (*N* = 5,630).

Subsequently, survival analyses using life table methods and log-rank tests showed that participants with low ASM mass (HR = 1.50, 95% CI = 1.29 ~ 1.76, *p* < 0.001), low PEFR (HR = 1.37, 95% CI = 1.14 ~ 1.64, *p <* 0.001), and probable RS (HR = 1.50, 95% CI = 1.27 ~ 1.78, *p* < 0.001) were more likely to develop CLDs earlier compared to those without these conditions (Figure 3). After adjusting for potential covariates (marital status, education level, residential area, smoking status, alcohol consumption, physical activities, CRP, hypertension, heart problems, digestive disease, rheumatism), Cox proportional hazard regression models further confirmed the significant influence of low ASM mass (HR = 1.47, 95% CI = 1.25 ~ 1.73, *p* < 0.001), low PEFR (HR = 1.31, 95% CI = 1.09 ~ 1.57, *p =* 0.004), and probable RS (HR = 1.49, 95% CI = 1.26 ~ 1.77, *p* < 0.001) on the earlier development of CLDs (Table 3). No interactions (*P* for interaction >0.05) between probable RS and potential confounders were observed through 7-year cohort (Figure 4).

**Table 3:** Cox regression model for 7-year incidence of chronic lung diseases, according to ASM mass, PEFR and probable RS (*N* = 5,630).

Variables	Model 1		Model 2	
	HR (95% CI)	*P*	HR (95% CI)	*P*
ASM mass (ref. normal to high)	1.47 (1.25 ~ 1.73)	**<0.001**	**–**	**–**
PEFR (ref. normal to high)	1.31 (1.09 ~ 1.57)	**0.004**	**–**	**–**
Probable RS (ref. no)	**–**	**–**	1.49 (1.26 ~ 1.77)	**<0.001**
Marital status (ref. married/married but separated)	1.20 (0.98 ~ 1.47)	0.082	1.22 (0.99 ~ 1.49)	0.059
Education level (ref. junior and below)	0.99 (0.76 ~ 1.29)	0.951	0.97 (0.75 ~ 1.26)	0.828
Residential area (ref. rural area)	1.11 (0.94 ~ 1.30)	0.214	1.10 (0.94 ~ 1.30)	0.226
Smoking status (ref. no)	1.41 (1.20 ~ 1.65)	**<0.001**	1.41 (1.20 ~ 1.66)	**<0.001**
Alcohol consumption (ref. no)	1.04 (0.88 ~ 1.22)	0.646	1.03 (0.88 ~ 1.22)	0.682
Physical activities (ref. no)	0.62 (0.40 ~ 0.95)	**0.028**	0.60 (0.39 ~ 0.92)	**0.019**
CRP (mg/L)	1.28 (1.08 ~ 1.51)	**0.004**	1.27 (1.08 ~ 1.50)	**0.005**
Hypertension (ref. no)	1.12 (0.95 ~ 1.31)	0.186	1.11 (0.94 ~ 1.30)	0.211
Heart problems (ref. no)	1.48 (1.21 ~ 1.80)	**<0.001**	1.48 (1.21 ~ 1.80)	**<0.001**
Digestive disease (ref. no)	1.48 (1.27 ~ 1.73)	**<0.001**	1.49 (1.27 ~ 1.74)	**<0.001**
Rheumatism (ref. no)	1.70 (1.47 ~ 1.96)	**<0.001**	1.70 (1.47 ~ 1.96)	**<0.001**

ASM, appendicular skeletal muscles; PEFR, peak expiratory flow rate; RS, respiratory sarcopenia; CPR, C-reactive protein; HR, hazard ratio; CI, confidence intervals. Model 1: ASM mass + PEFR + Covariates. Model 2: probable RS + Covariates. Bold values indicate statistically significant results (*P* < 0.05).

## Discussion

To the best of our knowledge, this is the first large-scale, population-based study to investigate the association of probable RS with CLDs among middle-aged and older Chinese adults. By integrating cross-sectional and longitudinal analyses, our findings indicated that both probable RS and its components (low ASM mass and low PEFR) were independently associated with an increased prevalence and incidence of CLDs. These associations persisted even after accounting for key covariates such as smoking status, biochemical indicators and comorbidities, further supporting the clinical significance of probable RS as a modifiable risk factor for the management of various CLDs.

The observed association between probable RS and an increased risk of CLDs likely stems from both mechanical and metabolic dysfunctions. First, reduced cough efficiency due to expiratory muscle weakness impairs airway clearance, potentially leading to mucus retention, bacterial colonization, and recurrent infections (21). These conditions may facilitate the development or progression of CLDs such as chronic bronchitis and chronic obstructive pulmonary disease (COPD) (22–24). Second, weakened respiratory muscles reduces ventilation efficiency, resulting in inadequate alveolar ventilation and hypoxemia. These abnormalities may further aggravate pulmonary vascular remodeling and fibrosis (25, 26). Moreover, decreased ventilatory reserve contributes to physical inactivity and exercise intolerance—both recognized as risk factors for CLDs risk and poor prognosis (27, 28). Third, probable RS may also lead to metabolic disturbances, particularly in lipid metabolism (29), as evidenced by the interaction between probable RS and hypolipidemia (*P* for interaction = 0.012) observed in our study. This interaction may synergistically create a pro-inflammatory environment through dysregulated fatty acid oxidation and increased mitochondrial reactive oxygen species (ROS) production. In turn, advanced CLDs may further accelerate muscle loss through glucocorticoid therapy, systemic inflammation, malnutrition, and reduced physical activity (30–32). In summary, these interconnected mechanisms form a vicious cycle—the muscle-lung axis—within which respiratory insufficiency and metabolic dysregulation jointly contribute to the development of CLDs.

In our analysis, PEFR and ASM showed a moderate positive correlation (*r* = 0.425), suggesting that they capture related yet distinct physiological domains. This supports the rationale for jointly incorporating both measures in defining probable RS. Notably, although both cross-sectional and longitudinal analyses suggest that probable RS may serve as a risk factor for various CLDs, the strength of the association differs substantially between the two approaches. Cross-sectional data indicated a 118% increased risk of CLDs in individuals with probable RS, whereas longitudinal analyses with an average follow-up of 7 years showed a more modest 49% increase. This discrepancy (118% vs. 49%) may be primarily attributable to the aforementioned vicious cycle of the “muscle-lung axis.” In cross-sectional studies, the simultaneous measurement of risk factors and disease outcomes increases the likelihood of reverse causality—i.e., pre-existing CLDs may contribute to muscle wasting or impaired pulmonary function, thereby exaggerating the observed association. Given that PEFR is a pulmonary function metric highly susceptible to current CLD status (33), its use in cross-sectional data may introduce greater reverse causality. Results based on PEFR (176% vs. 31%) provide further support for this hypothesis. However, the effect size of low ASM mass shows only a minor difference between the two study designs (79% vs. 47%), which is reasonable because ASM mass, as an indicator of muscle mass, typically changes gradually and is less affected by existing CLDs. Another potential explanation for the “118% vs. 49%” discrepancy is selective attrition in longitudinal studies, whereby high-risk individuals may die early or be lost to follow-up, leading to risk underestimation. In contrast, cross-sectional studies may overestimate associations by capturing long-term survivors, introducing survivor bias. Ingram and Kleinman’s analysis of NHANES I data supports these findings, noting that logistic models—especially those ignoring person-time—tended to yield higher estimates than Cox models due to the exclusion of survival time (34).

The current study offers multiple advantages. Firstly, the participants were drawn from a national longitudinal survey of community-dwelling adults, making generalizability of our results relatively high. Secondly, this is the first study to both cross-sectionally and longitudinally explore the relationship between probable RS, as determined by the Japanese Working Group for Respiratory Sarcopenia, and CLDs in an Asian context, while accounting for various confounding variables. Lastly, our findings contribute novel insights into lowering both the prevalence and incidence of CLDs by addressing probable RS among older adults living in communities.

However, the limitations of this study should be acknowledged. First, PEFR was used in our study as a surrogate marker for respiratory muscle strength due to its feasibility in large-scale epidemiological surveys. However, it can also be affected by airway obstruction from underlying CLDs and participant effort. As such, it may not fully capture true respiratory muscle strength, especially compared to more direct measures like maximal inspiratory pressure. This limitation should be acknowledged when interpreting the results. Nevertheless, our longitudinal analysis excluded individuals with baseline CLDs, likely minimizing confounding from pre-existing airway obstruction. Even after 7 years of follow-up, both lower PEFR and probable RS remained significantly associated with incident CLDs, supporting their predictive validity in identifying individuals at risk for CLDs. Second, the ASM mass in our analysis was calculated using an anthropometric formula, which may not exactly match the results from DXA or bioelectrical impedance analysis (BIA). This formula, created by Wen et al. (35) for the Chinese population, has shown an adjusted R^2^ of 0.90 and a standard error of 1.63 kg when compared to DXA. While random errors can occur, they tend to balance out in large-scale studies, making their impact on the overall findings minimal. Thus, this method, though seemingly simpler than DXA, remains practical and reliable, especially in resource-constrained environments. In fact, it has been validated in nearly 100 studies, further proving its reliability. Third, CLDs in our study were defined by self-reported physician diagnoses, a widely used method in large-scale epidemiological surveys such as CHARLS. While this approach is practical and cost-effective, it may introduce non-differential misclassification due to recall error or undiagnosed cases. Importantly, such non-differential misclassification typically biases effect estimates toward the null, thereby potentially underestimating the true associations between probable RS and CLDs (36). Consequently, the observed associations in our study may be conservative and the actual risks might be stronger than reported. Finally, while we incorporated several potential confounding factors such as age, smoking habits, and biochemical indicators into our multivariable analysis, some other relevant variables—such as history of respiratory infections and long-term exposure to air pollution—were not available in the CHARLS dataset and thus could not be adjusted for, which may have led to residual confounding.

## Conclusion

In conclusion, this study highlights the significant impact of probable RS on CLDs in community-dwelling middle-aged and older Chinese adults. This findings suggest that improving respiratory muscle health could not only enhance outcomes for individuals with existing CLDs, but also serve as a preventive measure for aging populations at risk of developing these diseases.

## Data Availability

The original contributions presented in the study are included in the article/Supplementary material, further inquiries can be directed to the corresponding author.
