# Supplementary figures and images for: Influence of probable respiratory sarcopenia on chronic lung diseases: a population-based cohort study of community-dwelling Chinese older adults
Source: Front Med (Lausanne). 2025 Aug 19;12:1617808. doi: 10.3389/fmed.2025.1617808 (PMC12403996; doi:10.3389/fmed.2025.1617808)

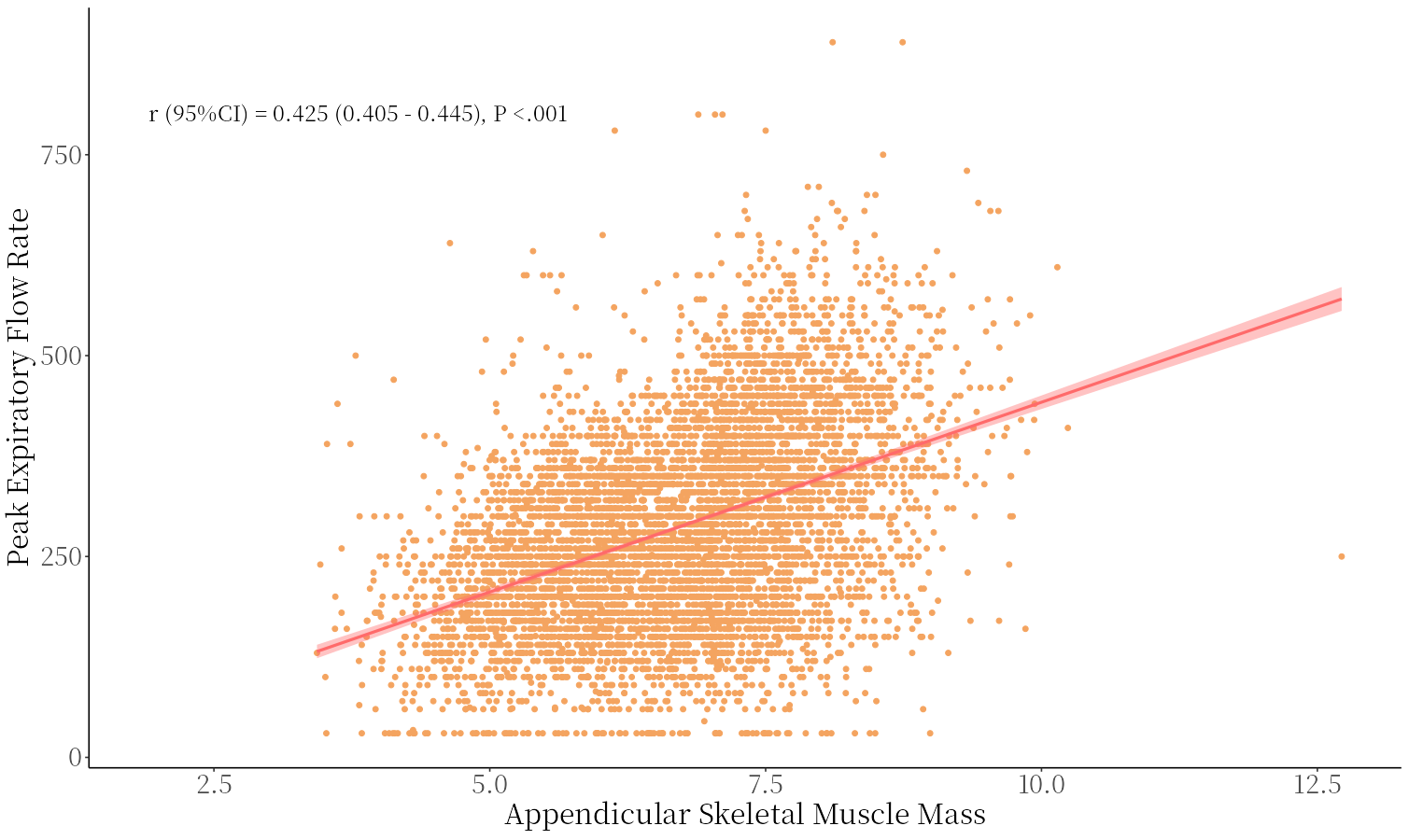

Supplement: Supplementary file 1 [file Image_1.PNG]
